# Network module detection: Affinity search technique with the multi-node topological overlap measure

**DOI:** 10.1186/1756-0500-2-142

**Published:** 2009-07-20

**Authors:** Ai Li, Steve Horvath

**Affiliations:** 1 Department of Human Genetics and Department of Biostatistics, University of California, Los Angeles, CA 90095, USA

## Abstract

**Background:**

Many clustering procedures only allow the user to input a *pairwise *dissimilarity or distance measure between objects. We propose a clustering method that can input a multi-point dissimilarity measure d(i1, i2, ..., iP) where the number of points P can be larger than 2. The work is motivated by gene network analysis where clusters correspond to modules of highly interconnected nodes. Here, we define modules as clusters of network nodes with high *multi-node *topological overlap. The topological overlap measure is a robust measure of interconnectedness which is based on shared network neighbors. In previous work, we have shown that the multi-node topological overlap measure yields biologically meaningful results when used as input of network neighborhood analysis.

**Findings:**

We adapt network neighborhood analysis for the use of module detection. We propose the Module Affinity Search Technique (MAST), which is a generalized version of the Cluster Affinity Search Technique (CAST). MAST can accommodate a multi-node dissimilarity measure. Clusters grow around user-defined or automatically chosen seeds (e.g. hub nodes). We propose both local and global cluster growth stopping rules. We use several simulations and a gene co-expression network application to argue that the MAST approach leads to biologically meaningful results. We compare MAST with hierarchical clustering and partitioning around medoid clustering.

**Conclusion:**

Our flexible module detection method is implemented in the MTOM software which can be downloaded from the following webpage:

## Findings

While most clustering procedures use a pairwise dissimilarity (distance) measure as input, we present a clustering procedure that can accommodate a multi-point dissimilarity measure *d*(*i*_1_, *i*_2_, ..., *i*_*P*_) where *P *> 1 is the number of points and the indices *i*_*k *_= 1, ..., *n *run over the *n *objects. Since we are mainly interested in a network application, we will refer to the objects as nodes and the corresponding measure as multi-node dissimilarity.

A multi-node (P-point) dissimilarity measure *d*(*i*_1_, *i*_2_, ..., *i*_*P*_) is defined to satisfy the following properties:

i) it takes on non-negative values, i.e.



ii) it equals 0 when all indices are equal, i.e.,



iii) it is symmetric with respect to index permutations, i.e.



Note that this definition reduces to that of a pairwise dissimilarity when *P *= 2.

Several approaches can be used to define a multi-node dissimilarity measure. For example, a pairwise dissimilarity measure *d*(*i*_1_, *i*_2_) gives rise to multi-node dissimilarity measure by averaging all pairwise dissimilarities, e.g. a 3 node measure can be defined as follows

(1)

The proposed module affinity search technique (MAST) can be used for any pairwise or multi-node dissimilarity measure. But our particular focus is the multi-node topological overlap measure (reviewed below) since it was found useful in network neighborhood analysis [1]. The topological overlap measure is defined for weighted and unweighted networks that can be represented by a (weighted) adjacency matrix *A *= [*a*(*ij*)], i.e. a symmetric similarity matrix with entries between 0 and 1. In an unweighted network, *a*(*ij*) = 1 if nodes *i *and *j *are connected and 0 otherwise. In a weighted network, 0 ≤ *a*(*ij*) ≤ 1 encodes the pairwise connection strength. Examples of such networks include gene co-expression networks and physical interaction networks.

A simple approach for measuring the dissimilarity between nodes *i *and *j *is to define *dissA*(*ij*) = 1 - *a*(*ij*). However, this measure is not very robust with respect to erroneous adjacencies. Spurious or weak connections in the adjacency matrix may lead to 'noisy' networks and modules [2]. Therefore, we and others have explored the use of dissimilarity measures that are based on common interacting partners or on topological metrics [3-7]. In this article, we will define modules as sets of nodes that have high topological overlap.

### Review of the pairwise topological overlap measure

The topological overlap of two nodes reflects their similarity in terms of the commonality of the nodes they connect to. Two nodes have high topological overlap if they are connected to roughly the same group of nodes in the network (i.e. they share the same neighborhood). In an unweighted network, the definition of the pairwise topological overlap measure in the supplementary material of [3] can be expressed in set notation as

(2)

where *N*(*i*_1_, *i*_2_) denotes the set of common neighbors shared by *i*_1 _and *i*_2_, *N *(*i*_1_, - *i*_2_) denotes the set of the neighbors of *i*_1 _excluding *i*_2 _and |·| denotes the number of elements (cardinality) of its argument. The Binomial coefficient  = 1 in the denominator of Eq. (2) is an upper bound of *a*(*i*_1_*i*_2_). One can show that 0 ≤ *a*(*i*_1_*i*_2_) ≤ 1 implies 0 ≤ *t*(*i*_1_*i*_2_) ≤ 1 [1,8,9], i.e. *t*(*i*_1_*i*_2_) can be considered a normalized measure of the number of shared direct neighbors. In the following, we will review how to extend this pairwise measure to multiple nodes.

### Review of the multi-node topological overlap measure

The topological overlap measure presented in Eq. (2) is a pairwise similarity measure. While pairwise similarities are widely used in clustering procedures, we have shown that it can be advantageous to consider multi-node similarity measures [1]. There are several ways to extend this measure to a multi-node similarity. The first extension (referred to as average pairwise extension) is to simply average the pairwise measure across all pairwise index selections as described in Eq. (1). The second extension (referred to as stringent MTOM extension) keeps track of the numbers of neighbors that are truly shared among multiple nodes. For example, the MTOM involving 3 different nodes *i*_1_,*i*_2_,*i*_3 _is defined as

(3)

where *N*(*i*_1_, *i*_2_, *i*_3_) is the set of the neighbors shared by *i*_1_, *i*_2 _and *i*_3 _and *N *(*i*_1_, *i*_2_, - *i*_3_) is the set of the neighbors shared by *i*_1 _and *i*_2 _excluding *i*_3_. The Binomial coefficient  = 3 in the denominator of Eq. (3) is the upper bound of *a*(*i*_1_*i*_2_) + *a*(*i*_1_*i*_3_) + *a*(*i*_2_*i*_3_) and equals the number of connections that can be formed between *i*_1_, *i*_2_, and *i*_3_. One can easily prove 0 ≤ *a*(*i*, *j*) ≤ 1 implies that 0 ≤ *t*(*i*_1_*i*_2_*i*_3_) ≤ 1. The stringent MTOM extension is related to the average pairwise TOM extension (Eq. 1) but it puts particular weight onto neighbors that are truly shared between the nodes. Below, we briefly compare of the two multi-node extensions (and corresponding dissimilarity measures).

### MTOM for weighted networks

To extend the MTOM measure to weighted networks, we express the set notation in terms of the entries of the adjacency matrix as follows

(4)

Note that these formulas remain mathematically well defined for a weighted adjacency matrix 0 ≤ *a*(*i*_1_*i*_2_) ≤ 1. Therefore, it is natural to use these algebraic formulas for defining the MTOM measure for a *weighted *networks. It is straightforward to extend the above equations for *P *= 3 nodes to more than 3 nodes, e.g. our MAST software can deal with any integer *P *> 1.

Since the topological overlap measure accounts for connections to shared neighbors, it is particularly useful in the context of sparse network, e.g. protein-protein interaction networks [3,8,10]. A comparison of the topological overlap measure to alternative measures (e.g. adjacency and correlation-based measures) can be found in [1,7].

### Comparison of MTOM based dissimilarity measures

The MTOM measures is a similarity measure that has an upper bound of 1. To turn it into a dissimilarity we subtract it from 1. For example, a two-node dissimilarity is given by



and a three node dissimilarity measures are given by

(5)

As outlined in Eq. (1), an alternative 3-node dissimilarity can be defined as follows

(6)

On real data, we find that the two 3 point dissimilarity measures (Eq. 5) and (Eq. 6) are highly correlated (*r *≈ .9) across index triplets with distinct indices, i.e. when *i*_1 _≠ *i*_2 _≠ *i*_3_. But if 2 indices are equal and different from the third index, (e.g. when *i*_1 _= *i*_2 _≠ *i*_3 _which entails *dissTOM *2(*i*_1_, *i*_2_) = 0) then the average pairwise dissimilarity (Eq. 6) takes on substantially lower values than dissimilarity (Eq. 5). Across all (possibly non-distinct) index triplets, we find a weak correlation (*r *≈ 0.3) between the two 3 point dissimilarity measures. In the following, we proceed with the non-pairwise dissimilarity measures (e.g. Eq. 5).

### Review of network neighborhood analysis using MTOM

In a previous publication, we have shown that the multi-node topological overlap measure (MTOM) can perform better than a pairwise measure in the context of network neighborhood analysis [1]. Since network neighborhood analysis is an important step in our proposed MAST procedure for module detection, we will review it in the following. Neighborhood analysis aims to find a set of nodes (the neighborhood) that is similar to an initial 'seed' set of nodes. Intuitively speaking, a neighborhood is composed of nodes that are highly connected to the given seed set of nodes. Since many of our applications involve networks comprised of genes, we will use the words 'node' and 'gene' interchangeably in this article. Neighborhood analysis facilitates a guilt-by-association screening strategy for finding genes that interact with a given set of biologically interesting genes. Informally, we refer to the resulting neighborhood as MTOM neighborhood. The MTOM-based neighborhood analysis requires as input an initial seed neighborhood composed of *S*_0 _≥ 1 node(s) and the requested final size of the neighborhood *S*_*t *_= *S*_0 _+ *S*, where *S *is the number of nodes that will be added to the initial neighborhood. For each node outside of the initial (seed) neighborhood, the MTOM software computes the MTOM value with the initial neighborhood. Next the *S *nodes with the highest MTOM values are selected and added to the initial seed neighborhood.

#### Computational Speed

In the following, we report computation times for carrying out MTOM neighborhood analysis on a computer with the following specifications: Intel Pentium 4 CPU 2.40 GHz 2.39 GHz, 1.00 G of RAM. To search for 10 neighbors of a gene, MTOM required 10 seconds in a network comprised of 2000 nodes, 2 minutes in a network of 5000 genes, 12 minutes in a network comprised of 10 k genes, and over 1 hour in a network comprised of 150 k genes.

### Finding modules (clusters) in a network

The detection of modules in a network is an important task in systems biology since genes and their protein products carry out cellular processes in the context of functional modules. Here we focus on module identification methods that are based on using a node dissimilarity measure in conjunction with a clustering method. We define modules as clusters of densely interconnected nodes. Although our method can be easily be adapted to any (dis-)similarity measure, our software implementation uses an extension of the topological overlap measure, which has been used in many network applications [1,3,7,8,11,12]. Numerous clustering methods exist for pairwise dissimilarity measures. We review hierarchical clustering and partitioning around medoids in more detail since we compare them to the proposed MAST procedure.

### Review of hierarchical clustering with the pairwise topological overlap measure

Before we describe the details of the MAST procedure, we method we first review a widely used module detection method which defines modules as branches of a hierarchical clustering tree. The standard approach for using the pairwise topological overlap measure for module detection is to use it as input of a hierarchical clustering procedure which results in a cluster tree (dendrogram) [13]. Next clusters (modules) are defined as branches of the cluster tree. Toward this end, one can use the R package dynamicTreeCut which implements several branch cutting methods [14]. Hierarchical clustering procedure has led to biologically meaningful modules in several applications [3,8,10,11,15-19], but it has one major limitation: it can only accommodate a *pairwise *dissimilarity measure. In contrast, our MAST procedure can accommodate a multi-node measure.

### Review of partitioning around medoid clustering

Partitioning around medoids (PAM, [13]), also known as k-medoid clustering, is a widely used clustering method, which generalizes k-means clustering. Compared to the k-means approach that is based on a Euclidean distance measure, PAM accepts any *pairwise *dissimilarity measure. The number *k *of clusters (partitions) is a user defined parameter. The PAM-algorithm iteratively constructs representative objects (medoids) for each of the *k *clusters. After finding a set of *k *initial medoids, corresponding *k *clusters are constructed by assigning each observation to the nearest medoid. After a preliminary clusters assignment, new medoids are determined by minimizing the within cluster dissimilarity measure. The procedure is iterated until convergence to a (local) minimum.

Similar to hierarchical clustering PAM can only accept a pairwise dissimilarity measure. Another limitation of PAM is that it does not allow for un-assigned (un-clustered) nodes. In many gene co-expression network applications, there are thousands of background genes that should not be forced into a module [8,14].

### Review of CAST

The Cluster Affinity Search Technique (CAST) [20] has been found to be an attractive clustering procedure for module detection in gene expression data. CAST is a sequential procedure that defines clusters one at a time. Since we have network applications in mind, we will refer to the clusters as modules. After detecting a module, CAST removes the corresponding nodes from consideration and initializes the next module. Module detection proceeds by adding and removing nodes based on a similarity measure between the nodes and the module members. A node is added if its similarity to the module exceeds a user-defined threshold. At each iteration the 'loosest' node will be dropped if its similarity to the module falls below the threshold. Note that the threshold is constant for the whole clustering procedure, which is why we refer to it as global threshold.

## Module Affinity Search Technique (MAST)

MAST extends the CAST procedure to module detection in a network. There are two major extensions. First, we propose to use a multi-node similarity measure instead of a pair-wise similarity since we have found evidence that a multi-node topological overlap measure (MTOM) can be more meaningful in network neighborhood analysis [1]. The second extension (described below) describes a local stopping rule for module growth that is used along the global stopping rule.

The starting point of the MAST procedure is an initial set of seeds for network neighborhood analysis (described above). These seeds can be user-defined or the procedure will automatically choose them based on their network connectivity *k*(*i*) = *sum*_*j *≠ *i*_*a*(*ij*). Since the automatic procedure uses the most highly connected nodes in a network as seeds, we often refer to the seeds informally as 'hub nodes'. Around these hub nodes, MAST defines an MTOM neighborhood of size 20 (referred to as hub neighborhood). We have found that the hub neighborhood size of 20 works well in our simulations and applications but this parameter value could be changed.

**Figure 1:**
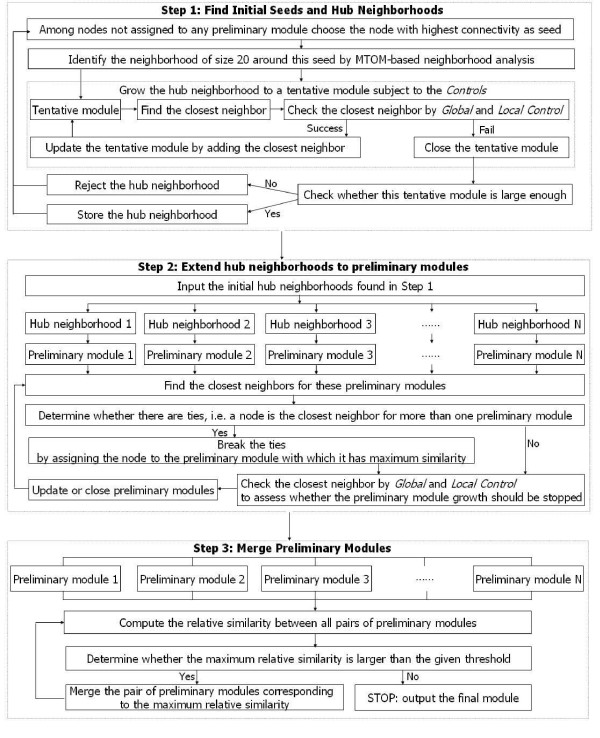
**Flowchart of the MAST approach**. The Figure outlines the steps of the MAST procedure. In step 1, the MAST procedure finds MTOM neighborhoods around initial seed nodes. The software allows the user to specify an initial set of seed nodes or, as alternative, we have also implemented an automatic procedure for picking seed nodes. In step 2 the hub neighborhoods are extended to preliminary modules. In this step, the initial hub neighborhoods comprised of 20 nodes are grown into larger modules in a stepwise fashion. The module growth is subject to stopping rules. In step 3, preliminary modules may be merged. Since some of the preliminary modules may be very similar, it can be advantageous to merge them into larger modules. The merging step is repeated until no pair of modules can be found with a relative between-module similarity larger than the threshold.

A flowchart of the MAST can be found in Figure 1. The procedure follows three steps. In step 1, it finds initial seeds (highly connected hubs) and their corresponding hub neighborhoods. Using neighborhood analysis, the hub neighborhoods are further extended to 'tentative modules'. If the tentative modules pass user specified control thresholds, they are grown into preliminary modules. In step 2, the hub neighborhoods are extended (grown) to modules in a simultaneous fashion. In step 3, similar modules are merged. Toward this end, the procedure assesses the relative similarity between the preliminary modules.

### MAST stopping rules

After a hub neighborhood has been identified, MTOM neighborhood analysis is used to grow a preliminary module around it. An important question is when to stop the module growth. Here we propose two stopping rules. At each step of the module growth, the closest neighbor to the module is identified based on the MTOM measure. The module growth proceeds until the closest neighbor falls below a global or a local control threshold. Both stopping rules for module growth consider a measure of relative similarity between a node and a module. We will make use of the following notation. Lower case indices denote individual nodes (e.g node *i*); upper case indices denote modules (e.g. module *K*). We denote the set of indices of nodes within module *K *by *C*(*K*). The module size (i.e., the number of nodes in module *K*) is denoted by *N*(*K*).

In the following, we describe measure for assessing a) the average similarity of module nodes, b) the similarity between a node and a module, and c) the similarity between two modules.

The pairwise similarity between nodes *i *and *j *is denoted as *s*(*i*, *j*). Below, we choose the pairwise topological overlap measure for *s*(*i*, *j*).

The within-module similarity *s*(*K*) of module *K *is defined as the average pairwise similarity between module nodes, i.e

(7)

Below, we will refer to this quantity informally as *module tightness*.

The average within-module similarity *S*_*W *_(*K, J*) for clusters *K *and *J *is the weighted average of the individual within-module similarities, i.e.,

(8)

The similarity *s*(*K, j*) between module *K *and node *j *is defined as the average pairwise similarity between node *j *and the module nodes, i.e.,

(9)

The relative similarity *r*_*Between*_(*K*, *i*) between module *K *and node *i *is defined as follows

(10)

The between-module similarity *S*_*Between*_(*K, J*) between modules *K *and *J *is defined as the average between module similarity, i.e.,

(11)

The relative similarity *R*_*Between*_(*K, J*) between modules *K *and *J *is defined as follows

(12)

The first stopping rule, referred to as *global control*, stops module growth when the relative similarity between the module and its closest neighbor falls below a global threshold. Analogous to its use in CAST, the global stopping rule is used to prevent the addition of nodes that do not exceed a minimum similarity threshold with regard to the module. The *Global Control *assesses whether the relative similarity *r*_*Between*_(*K*, *i*) between module *K *and the closest node *i *is less than a the global threshold.

**Figure 2:**
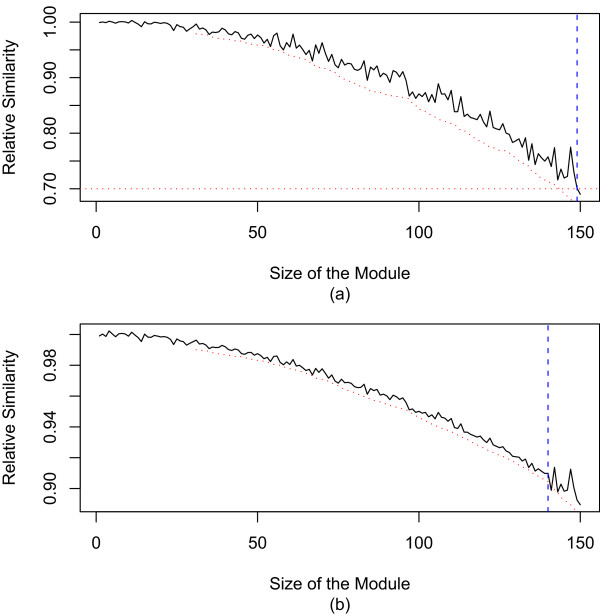
**Global control and local control of module detection**. The black curve shows the observed relative similarity *r*_*Between*_(*K*, *i*) (y-axis) between a module *K *and the *i*th closest node versus the growth history (x-axis). The red curve shows the lower bound of the prediction intervals from a local linear regression model. The red horizontal line shows the global threshold *T*_*global*_. According to the global control stopping rule, module growth stops when the black curve falls below *T*_*global*_. According to the local control stopping rule, module growth stops when the black curve falls below the red curve. a) Example where module growth stops because of global control with *T*_*global *_= 0.70. The dashed vertical line shows the final module size. b) Example where module growth stops because of a local control prediction interval with *T*_*local *_= 0.9999.

The second stopping rule, referred to as *local control*, considers the trend of the relative similarity measure as a function of the growth history, see Figure 2. Module growth is stopped if the direction of the trend falls below a local threshold. The local stopping rule is used to prevent the addition of nodes that would lead to a strong discontinuity in the module growth history (as reflected by the pattern of relative similarities). The local stopping rule uses the relative similarities of previous nodes to prevent the addition of outlying nodes that do not fit the trend. To illustrate the local stopping rule, assume that the relative similarity of each added node was 1 percent smaller than that of the previous node. If the relative similarity of the next node under consideration is say 30 percent smaller than that of the previous node, it does not fit the trend. Adding this node would lead to a severe discontinuity in the module growth history and lower the 'admissions standard' set by the previous nodes. Since this outlying node may be part of a different module, it is not added to the module. Specifically, the *Local Control *assesses whether the relative similarity *r*_*Between*_(*K*, *i*) of the closest node *i *does not fit the trend of growth history of module *K*. Specifically, denote by {*r*_*Between*_(*K*, *a*_1_), *r*_*Between*_(*K*, *a*_2_), ..., *r*_*Between*_(*K*, *a*_*N*(*K*)_)} the set of relative similarities according to the module growth history, i.e., the relative similarities are ordered according to when the corresponding members were added to the module. The module *K *grows at each step. To estimate the trend, a local 'window' of *w *most recent nodes is considered. To estimate the trend a linear regression model is fit to the vector of *w *most recent relative similarities {*r*_*Between*_(*K*, *a*_*N*(*K*)-*w*+1_), *r*_*Between*_(*K*, *a*_*N*(*K*)-*w*+2_), ..., *r*_*Between*_(*K*, *a*_*N*(*K*)_)}, versus the vector {1, 2, ..., *w*}. This (local) univariate linear regression model can be used to estimate a prediction interval of the relative similarity for the next candidate module member. Based on the regression model, node *j *fails the *Local Control *if *r*_*Between*_(*K*, *j*) is smaller than the lower bound of the prediction interval. In our applications, we use a very wide prediction interval (default *T*_*local *_= 0.9999) but this proportion is a user-defined threshold. Figure 2 illustrates the two stopping rules.

### Steps of the MAST procedure

In the following, we outline the steps of the MAST procedure in more detail.

#### Step 1, find hub neighborhoods

The MAST procedure is based on finding MTOM neighborhoods around initial seed nodes. The software allows the user to specify an initial set of seed nodes or, as alternative, we have also implemented an automatic procedure for picking seed nodes. Since each node is part of a network, one can compute its connectivity (degree) to other nodes: *k*_*i *_= Σ_*j *≠ *i *_*a*(*ij*). We have found that seeds with high connectivity tend to produce more meaningful neighborhoods [1]. Therefore, the first seed is chosen as the most highly connected 'hub' node in the network.

Next we use MTOM neighborhood analysis to arrive at a tight neighborhood around the hub node. We refer to the result as *hub neighborhood*. The default hub neighborhood size of 20 can be changed by the user. Using the stopping rules described above, the hub neighborhood is then grown to a preliminary module. If the size of the preliminary module is below a user-defined threshold, the initial hub node is rejected and the next most highly connected node will be chosen as seed. But if the size of the preliminary module passes the threshold, then the preliminary module nodes will not be considered when choosing a second seed. Thus, the second seed will be the most highly connected node outside the first preliminary module. This process of choosing seeds is repeated until all nodes have been considered.

#### Step 2, extend the hub neighborhoods to preliminary modules

In this step, the initial hub neighborhoods comprised of 20 nodes are grown into larger modules in a stepwise fashion. We have implemented a competitive module growth process where the modules compete for the remaining nodes. At each step, the algorithm finds the closest MTOM neighbor of each module. If a node is the closest neighbor for 2 or more modules, it will be assigned to the module with which it has the highest MTOM value. The module growth is subject to the stopping rules described above.

#### Step 3, merge the preliminary modules

Since some of the preliminary modules may be very similar, it can be advantageous to merge them into larger modules. The merging procedure computes the maximum relative between-module similarity  across all possible pairs of modules. If the maximum is above a user-defined threshold *T*_*merge*_, the corresponding modules will be merged. The merging step is repeated until no pair of modules can be found with a relative between-module similarity larger than the threshold.

### Parameter Settings

As outlined above, the MAST procedure allows the user to specify several parameters. Changing the parameters will lead to different modules. We find that the resulting modules are highly dependent on the initial seeds. While we allow the user to specify initial module seeds, we have implemented an automatic seed detection method in step 1. Other parameters are the threshold for the global control *T*_*global *_(step 1 and step 2), the threshold for the local control *T*_*local *_(step 2), the minimum module size, and the merging threshold *T*_*merge*_. The software uses the following default settings *T*_*global *_= 0.6, *T*_*local *_= 0.9999, and minimum module size of 50. In our applications, we illustrate how the results change as a function of different parameter settings. In general, the higher *T*_*global *_and the lower *T*_*local*_, the tighter will be the resulting modules.

## Application to a gene co-expression network

The MAST procedure can be used to find clusters (modules) in any undirected network (e.g. protein-protein interaction networks). But we will focus here on the analysis of a weighted correlation network (also known as weighted gene co-expression network) [8,12,21,22]. An undirected network is fully specified by its *adjacency matrix a*(*ij*), a symmetric *n *× *n *matrix with entries in [0, 1]. The adjacency *a*(*ij*) encodes the network connection strength between nodes *i *and *j*. To calculate the adjacency matrix, an intermediate quantity called the *co-expression similarity s*(*i*, *j*) is first defined. The default method defines the co-expression similarity *s*(*i*, *j*) as the absolute value of the correlation coefficient between the profiles of nodes *i *and *j*:



but alternative measures (e.g. based on mutual information or more robust correlation measures) could also be used. Using a thresholding procedure, the co-expression similarity is transformed into the adjacency. An unweighted network adjacency *a*(*ij*) between gene expression profiles *x*_*i *_and *x*_*j *_can be defined by hard thresholding the co-expression similarity *s*(*i*, *j*) as

(13)

where *τ *is the hard threshold parameter. While unweighted networks are widely used, they do not reflect the continuous nature of the underlying co-expression information and may thus lead to an information loss. In contrast, weighted networks allow the adjacency to take on continuous values between 0 and 1. A weighed network adjacency can be defined by raising the co-expression similarity to a power [8,21]:

(14)

with *β *≥ 1. The adjacency in Equation (14) implies that the weighted adjacency *a*(*ij*) between two genes is proportional to their similarity on a logarithmic scale, *log*(*a*(*ij*)) = *β *× *log*(*s*(*i*, *j*)).

### Comparing modules to known gene ontologies

We applied the MAST procedure to find modules in a subset of a brain cancer gene co-expression network [8,12]. The gene co-expression network was constructed on the basis of 55 brain cancer microarrays. A weighted adjacency matrix was defined as follows *a*(*ij*) = |*cor*(*x*_*i*_, *x*_*j*_)|^4 ^where *x*_*i *_and *x*_*j *_are the expression profiles of gene *i *and *j*, respectively. Our findings remain largely unchanged with regard to different choices of the power *β *= 4. To be able to relate modules to known gene ontologies, we restricted the analysis to the 520 genes with the following ontologies: apoptosis (205 genes), neurogenesis (229 genes) and DNA replication (86 genes). To assess the performance of different module detection methods, we compared module membership to the 3 known gene ontologies. We used the Rand index to measure agreement between the resulting modules and the gene ontologies. The Rand index is a widely used measure to evaluate the agreement between two partitions [23].

**Figure 3:**
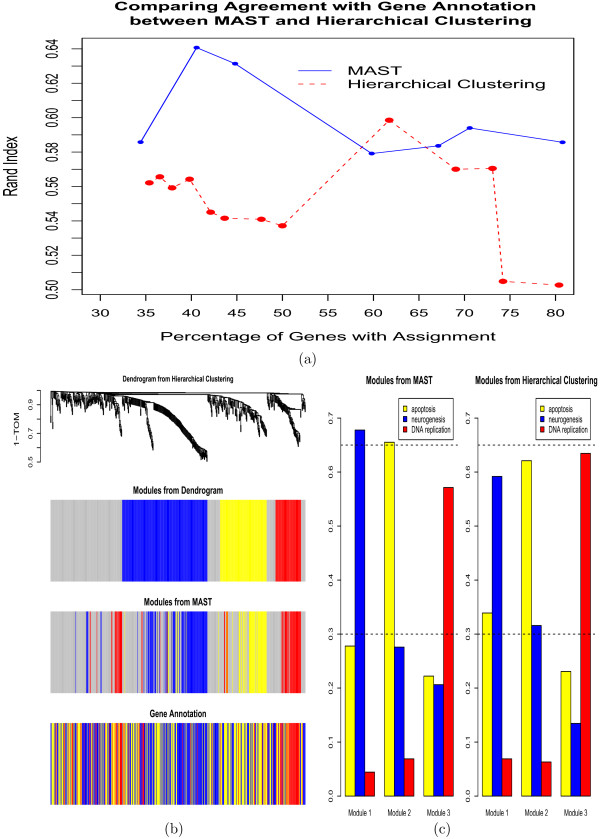
**Cancer gene network: comparing MAST to hierarchical clustering**. Both procedures allow for unassigned (unclustered) genes. (a) Rand index (y-axis) with gene ontology as a function of the percentage of assigned genes (x-axis). Since the blue line (MAST procedure) tends to be higher than the red line (hierarchical clustering), MAST performs better in this application. (b) Hierarchical clustering dendrogram with the pairwise TOM. The first color band underneath the tree shows the results of defining modules as branches of the tree. 'Grey' is reserved for unassigned genes. The second color band depicts the module assignment of the MAST procedure. The third colorband depicts the known gene ontologies. (c) The barplots on the left hand side and on the right hand side show the functional enrichment of modules (x-axis) found by the MAST procedure and by hierarchical clustering, respectively. The height of the barplots equals the proportion of module genes that are known to be apoptosis related (yellow bars), neurogenesis related (blue bars), and DNA replication related (red bars). Both clustering procedures find 3 similar modules.

We considered 3 different module detection methods: i) our MAST approach, ii) average linkage hierarchical clustering, and iii) Partitioning Around Medoids. All three procedures depend on input parameters. For example, the modules based on hierarchical clustering depend on the height cut-off used for cutting off branches. Partitioning around medoids uses the number of modules *k *as input parameter. For PAM, the Rand index ranges from 0.483 to 0.594. For hierarchical clustering, the maximum Rand index equals 0.598, see 3. For the MAST procedure, the maximum observed Rand index equals 0.64. Overall, we find that MAST performs best on these data. This can be seen from Figure 3 where we compare MAST with hierarchical clustering. To allow for a fair comparison between these two procedures, we report the Rand index as a function of the proportion of unassigned genes.

## Simulation studies

### Simulation I

Here we present a simple simulation study for highlighting the differences between MAST, PAM, and average linkage hierarchical clustering. We do not claim that the simulated example represents a good approximation to real gene expression data. A more complex model is described below.

For this simple example, we simulated 500 variables (features) that form 4 modules corresponding to sets of highly correlated variables. Specifically, we simulate 500 variables with values in 60 observations. The resulting data can be represented by a matrix {*x*_*ij*_} with 500 rows (1 ≤ *i *≤ 500) and 60 columns (1 ≤ *j *≤ 60). The variables were simulated to form 4 modules with sizes *n*_1 _= 100, *n*_2 _= 100, *n*_3 _= 150 and *n*_4 _= 150, respectively. For the *k*th module, we simulated a module seed vector *m*^*k *^and simulated module members (variables) around it. The *j*th value of the *i*th variable in the *k*th module was simulated as follows



where the stochastic noise  was simulated to follow a normal distribution with mean 0 and variance 0.25. We simulated the first three seeds to be highly correlated with the fourth seed. Thus, the fourth resulting module is mixed with the first three modules. Specifically, the simulated true seed variables  are given by



where the indicator function *I*(*condition*) equals 1 if the condition is true and 0 otherwise.

**Figure 4:**
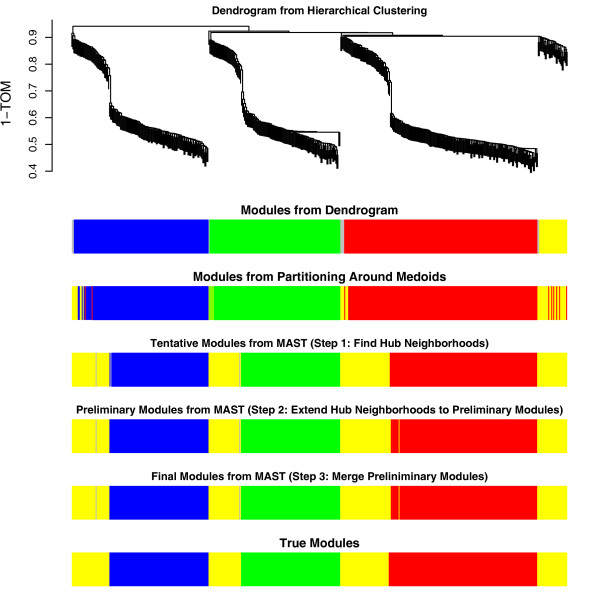
**Comparison of different module detection methods for simulation I with mixed modules**. The clustering tree results from average linkage hierarchical clustering with the pairwise topological overlap measure. The first color band underneath the tree colors the branches (modules) that result after cutting off the branches with a fixed height cut-off. When compared to the last color band that corresponds to the true simulated modules, it becomes clear that the method fails at retrieving the fourth (yellow) module irrespective of the height cut-off. The second color band corresponds to the module colors retrieved from PAM clustering with *k *= 4. It fails to retrieve true membership as one can see by comparing it to the true colors in the last row. Color bands 3 through 5 correspond to the steps of the MAST procedure. The fifth row shows the final result of the MAST procedure. When compared to the last color band that corresponds to the true simulated modules, it becomes clear that the MAST procedure retrieves the true underlying module structure. In particular, it can retrieve the yellow module as well.

To arrive at a weighted network (adjacency matrix), we first correlated the 500 variables *x*_*i *_with each other across the observations. This resulted in a 500 × 500 dimensional correlation matrix. To emphasize high correlations at the expense of low, correlations, we raised the entries of the correlation matrix to a power *β *= 2, i.e., *a*(*ij*) = |*cor*(*x*_*i*_, *x*_*j*_)|^2 ^where *x*_*i *_and *x*_*j *_are the variable vector across the 60 observations. We study whether the MAST procedure correctly retrieves the known underlying module membership in the simulated 4 modules. In Figure 4, we color the nodes of the first three modules by red, blue, and green respectively. The fourth module, which is highly correlated with the other modules, is colored in yellow. Figure 4 shows the MAST procedure retrieves the true modules correctly. It outperforms hierarchical clustering method and PAM when both use the pairwise TOM as input.

### Simulation Study II

Here we provide a more complex simulation study. Similar to simulation study I, we specify seed genes that lie at the center of the module. We simulated 5 modules. The seeds of the first two modules *m*^(1) ^and *m*^(2) ^correspond to vectors whose entries were randomly chosen to be 0 or 1. We added noise from a standard normal distribution *N*(0, 1). The seed of modules 3 was given by *m*^(3) ^= *a *× *m*^(1) ^+ *ϵ*_3 _where the coefficient *a *controls the dependence between the seeds and *ϵ *represents standard normal noise.

**Figure 5:**
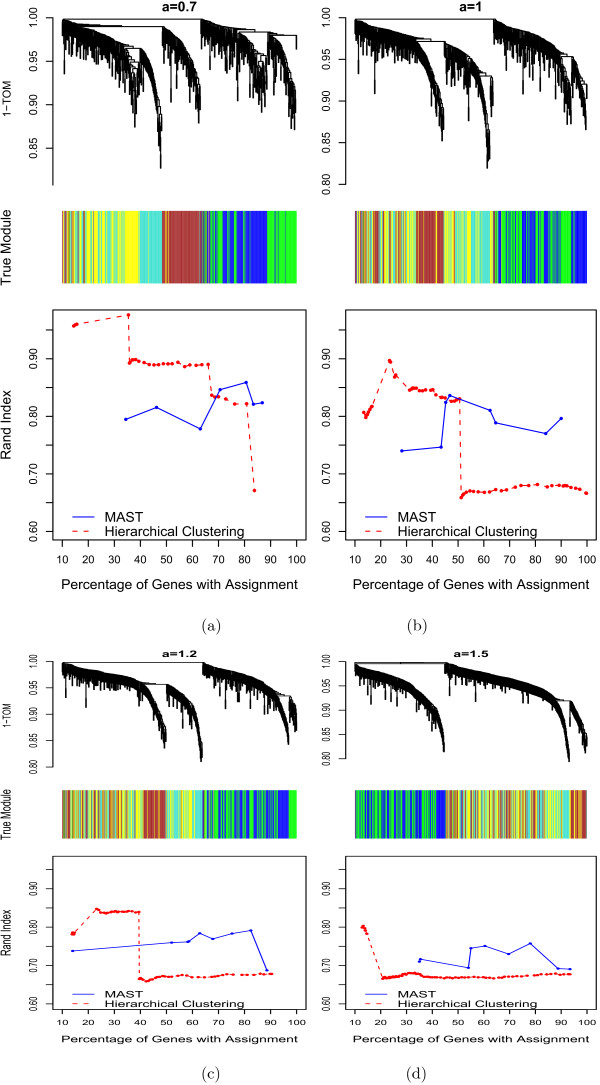
**Comparison of different module detection methods for simulation II**. Figures a) through d) correspond to modules of increasing dependency. The clustering trees correspond to average linkage hierarchical clustering with the pairwise topological overlap measure. The first color band underneath each clustering tree color-codes true simulated module membership. The plot under the color band shows the performance of the MAST procedure (blue line) as a function of different MAST parameters. The y-axis reports the Rand index versus the percentage of genes that were assigned to a module (x-axis). The red curve in the plot corresponds to the Rand index of the hierarchical clustering procedure that defines modules as branches. Note that when there is a strong dependence between the modules (Figure d), the MAST procedure outperforms hierarchical clustering.

Analogously, we chose the seeds of modules 4 and 5 to depend on seed 2, i.e., *m*^4 ^= *a *× *m*^(2)^+ *ϵ*_4_, m^(5) ^= *a *× *m*^(2) ^+ *ϵ*_5_. We simulated gene expression profiles around each of seeds with different correlations with the seed. The simulated gene expressions were chosen such that their correlation with the seed ranged from *r*_*min *_= 0.80 to 1. By increasing the parameter *a *one increases the dependency between the module seeds. We considered 4 scenarios corresponding to *a *= 0.7, *a *= 1.0, *a *= 1.2 and *a *= 1.5, respectively. For each of the 4 scenarios, we compared hierarchical clustering to the MAST procedure. As described above, both procedures depend on several input parameters. To allow for a fair comparison between hierarchical clustering and the MAST procedure, we report the Rand index as a function of the proportion of unassigned genes in Figure 5. Note that the red line corresponding to hierarchical clustering varies greatly while the blue line corresponding to the MAST procedure varies less. Thus, the the performance of the MAST procedure is more robust to parameter specification than that of hierarchical clustering. Figures 5b)-d) show that the MAST procedure outperforms hierarchical clustering as the dependence (similarity) between the modules increases. When there is a low dependence between the modules (*a *= 0.7, Figures 5a), we find that hierarchical clustering performs better than MAST when one tolerates a large number of un-assigned genes. But if the parameters of both procedures are chosen such that few genes are unassigned then the MAST procedure performs better.

Here we simulated data with a handful of modules. To simulate more realistic gene expression data involving an arbitrary number of modules, future studies could make use of the R function *simulateDatExpr *in the WGCNA R package [21].

## Conclusion

It is difficult to argue that one clustering procedure is better than another due to the lack of universally accepted benchmark data and a gold standard. We present simulation studies that illustrate that the proposed MAST procedure can be superior to hierarchical clustering and partitioning around medoids when dealing with simulated cluster data whose modules are generated around 'seed' nodes. In this situation, seed-based clustering method such as MAST are expected to perform well. Future comparisons should evaluate MAST on simulated cluster data that are not seed-centered.

Numerous clustering procedures have been proposed for the analysis of gene expression data [24-26]. Bi-clustering procedures cluster genes and samples simultaneously [27,28]. Here we propose a clustering method that can be used for a multi-node dissimilarity measure. The proposed MAST procedure is well suited for the use of any multi-node dissimilarity measure. In our application and software, we use the multi-node topological overlap measure and the intuition of network neighborhood analysis described in [1] but it is straightforward to adapt the MAST procedure to any other multi-node measure. For example, by averaging a pairwise dissimilarity measure, one can easily define a multi-node measure as described above. Therefore, the MAST procedure can also be used for any *pairwise *dissimilarity measure.

Using a gene co-expression network application and several simulated examples we provide evidence that the MAST procedure can outperform hierarchical clustering and PAM. But a more comprehensive comparison with alternative methods is desirable. Several procedures for finding modules in networks have been proposed, e.g. Cytoscape implements several procedures [29]. MAST can be used to find modules in gene expression data or other quantitative data after a correlation network is defined between the quantitative variables. When applied to a network (specified an adjacency matrix), the MAST procedure yields clusters of tightly interconnected modules. Since module sizes and module tightness can vary greatly in practice we have found it useful to implement an adaptive local stopping rule that considers the module growth history.

Our preliminary data (simulations and a co-expression network analysis) are encouraging but more comprehensive comparisons are needed. Our software implementation should facilitate an evaluation and comparison with other methods.

## Availability and requirements

The MAST procedure is implemented in the MTOM software. Project name: MTOM Software

Project home page: 

Operating system(s): Windows

Programming language: C

Licence: GNU GPL 3

## Competing interests

The authors declare that they have no competing interests.

## Authors' contributions

Both authors jointly developed the methods and wrote the article. Both authors read and approved the final manuscript.
